# Evolutionary history of *Plasmodium vivax* and *Plasmodium simium* in the Americas

**DOI:** 10.1186/s12936-022-04132-7

**Published:** 2022-05-03

**Authors:** Virginie Rougeron, Josquin Daron, Michael C. Fontaine, Franck Prugnolle

**Affiliations:** 1 International Research Laboratory, REHABS, CNRS-NMU-UCBL, George Campus, Nelson Mandela University, George, South Africa; 2 Laboratory MIVEGEC, University of Montpellier, CNRS, IRD, 900, rue Jean-François Breton, 34900 Montpellier, France; 3 Groningen Institute for Evolutionary Life Sciences (GELIFES), University of Groningen, Groningen, The Netherlands

**Keywords:** *Plasmodium vivax*, Evolutionary origin, Adaptation, Americas, Population genomics

## Abstract

Malaria is a vector-borne disease caused by protozoan parasites of the genus *Plasmodium*. *Plasmodium vivax* is the most prevalent human-infecting species in the Americas. However, the origins of this parasite in this continent are still debated. Similarly, it is now accepted that the existence of *Plasmodium simium* is explained by a *P. vivax* transfer from humans to monkey in America. However, many uncertainties still exist concerning the origin of the transfer and whether several transfers occurred. In this review, the most recent studies that addressed these questions using genetic and genomic approaches are presented.

## Background

*Plasmodium* parasites are the agents responsible for malaria, one of the worst scourges of mankind, with almost 300,000 infant deaths and about 200 million new clinical cases reported each year [1]. This infectious disease is the result of the long and complex co-evolutionary relationships of three main actors: a vertebrate host, a vector of the genus *Anopheles,* and a *Plasmodium* parasite. Among the seven *Plasmodium* species known to infect humans, *Plasmodium vivax* is the most prevalent human malaria parasite outside sub-Saharan Africa [2]. *P. vivax* infection causes severe clinical symptoms with significant effects on human health [3]. Moreover, the periodicity of *P. vivax* transmission confers only transient immune protection. Infected individuals present episodes of fever that may occur each month, leading to education delays in infected children and to direct economic consequences caused by the loss of productivity and income in infected adults [4]. Moreover, the emergence of therapeutic resistance and the description of severe and even fatal cases due to *P. vivax* question the benign status of vivax malaria [5]. Despite the fact that *P. vivax* is now considered as a major public health issue, the evolutionary history and adaptation of this parasite is still insufficiently understood.

Studying the evolutionary history of a pathogen is fundamental to obtain insights into the genetic variation involved in its adaptation, and into its geographic origin, distribution and migration. From an evolutionary point of view, it is now acknowledged that *P. vivax* history is characterized by a succession of several colonization events in different primate species and also in different human populations worldwide. *P. vivax* is present in almost all tropical and inter-tropical regions. These regions were colonized by this parasite more or less recently following the human migrations. The American continent was one of the last parts of the world to be colonized by modern human populations, approximately 15,000 years ago through the bridge that connected Alaska and Siberia [6]. For thousands of years, during their migrations to the Americas from all around the world, human populations carried with them several pathogen species that became endemic in the New World. This was the case, for instance, of *Plasmodium falciparum*, the most virulent malaria species, that was introduced in the Americas from Africa during the transatlantic slave trade between the sixteenth and nineteenth centuries [7]. In the Americas, malaria is now caused mainly by *P. vivax* (77.5% of human infections), followed by *P. falciparum* (20.8%) and *Plasmodium malariae* (2%) [8]. On the basis of the current knowledge, *P. vivax* was introduced in the Americas at least through two main colonization waves (in the pre-Columbian era and/or during the colonization by European countries) during which *P. vivax* infected new human populations (Amerindian and/or European populations) and new local vector species. Moreover, a recent transfer of *P. vivax* from humans to American monkeys gave rise to a new species that is genetically very close to *P. vivax*, and was named *Plasmodium simium*. How, through which routes, and when *P. vivax* arrived in the New World and became adapted to these new conditions are still controversial and ongoing questions.

In this review, the most up to date data about (i) the evolutionary histories of *P. vivax* and *P. simium* in the Americas, (ii) and their genetic adaptation in these new environments are presented.

### A still unclear evolutionary origin of *P. vivax* in the Americas

The most recent population genetic studies have supported the hypothesis that the current *P. vivax* populations across the world originated from Asia, and probably spread to African countries [9–11]. Conversely, the origin and demographic history of *P. vivax* populations in the Americas have received limited attention, and their origin, routes of migration, and time of colonization are still debated. Several approaches have been used to retrace *P. vivax* history in the New World. The most direct is to search for evidence of infection in archaeological human remains using molecular and immunological methods, which is a difficult task with pathogens. The second type of approach is indirect, through the analysis of the genealogical relationships between parasite populations using population genetic and genomic tools.

### Evidence of *Plasmodium vivax* parasites in American archaeological remains

Tito et al. [12] were among the first to assess whether ancient American populations were infected by *Plasmodium* parasites using metagenomic sequencing analysis of archaeological human faecal specimens. Their objective was to establish the microbiome composition of 1300-year-old paleo-faeces from two humans collected at the Cueva de los Muertos Chiquitos archaeological site (Durango, Mexico) using high-throughput sequencing methods (454/Roche GE-FLX pyrosequencing). They obtained about 45,000 shotgun DNA reads that did not matched any bacteria using BLAST, but could be mapped to the sequences of commensal and pathogenic organisms, including *Plasmodium* parasites [12]. They found that 34 reads matched sequences belonging to the *Plasmodium* genus (> 80% of identity, based on the BLASTN analysis). Unfortunately, they could not identify the *Plasmodium* species [12]. Ten years later, another study described the detection of *Plasmodium* in human coprolites collected in Puerto Rico (Vieques) dating approximately 1400 to 1700 before present [13]. The shotgun metagenomic sequencing strategy allowed identifying 127 reads that mapped to the *Plasmodium* genome with a mean identity of 81%. Nevertheless, these results need to be taken with caution because reads could be matched with different prokaryotes and eukaryotes species, even if reads matched with a higher assigner score to the *Plasmodium* query (and not a single assigner score with multiple *Plasmodium* species) [13]. If these reads are real evidence of *Plasmodium* DNA, these results suggest that *Plasmodium* parasites were probably already circulating in the American continent during the pre-Columbian era or arrived with the pre-Colombian human migrations, approximately 15,000 years ago when the first humans crossed the Siberia-Alaska bridge, or more recently, for instance, during the transpacific migration. Unfortunately, the exact *Plasmodium* species present in these paleo-faeces could not be identified, and the low-quality reads are not sufficient to draw any clear conclusion.

Gerszten et al. [14] provided the first immunological and histological evidence of *P. vivax* circulation in the Americas. This study investigated diseases contracted by pre-Columbian Inca populations, thus before the arrival of the European colonists in the sixteenth century. They collected several human mummies (dated between 3000 and 600 years before present) in cemeteries of civilizations originating from Southern Peru (Ice Valley) and Northern Chile (Azapa Valley). Paleo-pathologists could isolate 155 livers and spleens from these mummies that were screened for the presence of various infectious pathogens, including *Plasmodium* parasites. ELISA in situ hybridization, using antibodies against *P. vivax* and *P. falciparum*, showed that 67% of the tested specimens were positive for *P. vivax*, but none for *P. falciparum* [14]. Histological analysis of tissue sections after haematoxylin and eosin staining indicated that malaria pigment (haemozoin) was present in 30% of the tested samples. Moreover, these samples were *P. vivax*-positive by ELISA. On the basis of the absence of *P. falciparum* detection in these remains, the authors suggested that *P. falciparum* was imported in the Americas during the first transatlantic exchanges with Europe and Africa in the sixteenth century. Further, the high *P. vivax* detection rate in these samples suggested that *P. vivax* had been present in the Americas at least since the pre-Columbian era (i.e. before the fifteenth and sixteenth centuries) [14]. Despite the importance of such results to understand *P. vivax* evolutionary origin, some limitations must be acknowledged. First, Gerszten et al. [14] did not specify which antibodies were used for the ELISA assays. Second, they did not test the antibody cross-reactivity to confirm that the used antibodies were specific only for *P. vivax*. Indeed, the anti-*P. vivax* antibodies did not cross-react with *P. falciparum*, but they might have recognized other *Plasmodium* species present on the American continent, such as *Plasmodium malariae*.

To firmly confirm the presence and circulation of *P. vivax* parasites during the pre-Colombian era, future studies should focus on the analysis of more mummified tissues (e.g. liver or spleen), and on trying to PCR amplify *Plasmodium* conserved genes [e.g. several mitochondrial genes, the circumsporozoite (*CSP*) gene, or the merozoite surface protein (*MSP*) gene] that might allow validating the presence of *Plasmodium* parasites and more importantly the identification of the *Plasmodium* species.

### Population genetic approaches to retrace *P. vivax* history in the Americas

Several studies have tried to determine the origin of *P. vivax* in the Americas, through the analysis of gene polymorphisms, apicomplexan or mitochondrial genome phylogenies, and highly polymorphic microsatellite marker analysis.

The first important point is that the Americas are considered to be the last land colonized by the contemporaneous populations of *P. vivax* parasites through human migrations. This conclusion comes from the comparison of the population genetic characteristics of the parasite populations from South America and from other parts of the world. A colonization event is often associated with a founder effect: a rapid loss of genetic diversity and an increase of linkage disequilibrium (LD) due to the fact that the new populations are formed by only a small number of individuals from the source population. Therefore, populations resulting from recent colonization events are expected to have lower genetic diversity and higher LD compared with more ancient populations or the source populations [15, 16]. Imwong et al. [17] analysed nine microsatellite markers in 345 *P. vivax* populations from Asian and American genotypes and found that LD was higher in the American *P. vivax* populations. This suggests that the American *P. vivax* populations may be more recent than the Asian ones. Similarly, Rougeron et al. [9] genotyped and analysed 834 *P. vivax* isolates from around the world using 14 microsatellite markers and found that American *P. vivax* isolates had the highest LD and the lowest allelic richness [9]. These observations are also in favour of the hypothesis that the Americas were the last areas of the world colonized by *P. vivax*. Culleton et al. [18] compared the mitochondrial genome diversity of *P. vivax* parasites from Africa and from other areas of the world, and observed the lowest genetic diversity in American *P. vivax* populations. This again suggests that the New World would be the most recently colonized place by this parasite species [18]. Similar findings (lowest genetic diversity and/or highest LD in American *P. vivax* populations compared with the rest of the world) were obtained in population genomic analyses [10, 19], again suggesting that American regions were the last to be colonized by this parasite species, as is the case for *P. falciparum* [10, 19].

Another way to determine whether the American continent was the last place colonized by *P. vivax* is to calculate the time to the most recent common ancestor (TMRCA) in different populations. For instance, in a study on 282 sequences with 93 haplotypes covering the known geographical distributions of *P. vivax*, [20], American *P. vivax* samples displayed the shortest TMRCA. Another more recent population genomic study obtained the same result, with the *P. vivax* from America characterized by the shortest TMRCA [10]. This, again, is in favour of a more recent introduction of malaria to this continent [20]. All these studies indicate that the American continent was the last one to be colonized by *P. vivax* during its evolutionary history.

**Fig. 1:**
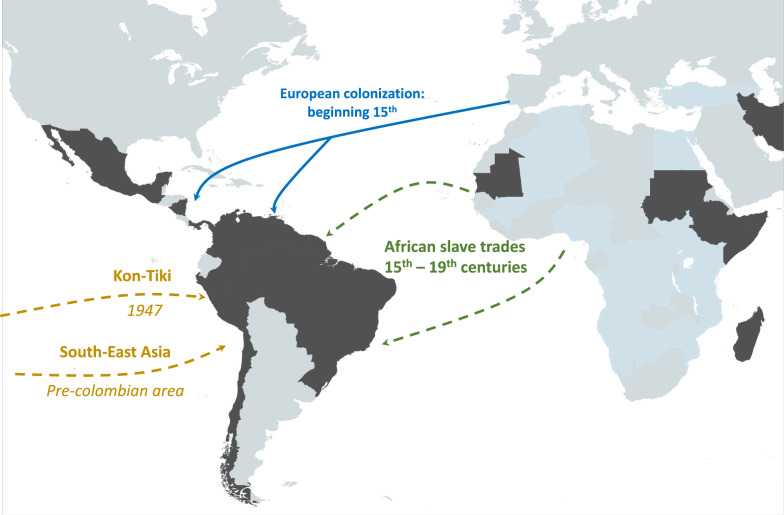
Schematic representation of the hypothetic origins of American *P. vivax*. The main routes of the transatlantic slave trades (in green), major European colonization routes between the fifteenth and nineteenth centuries (in blue), and Asian migration waves (in brown-yellow) are indicated. When the migration route and origin have been confirmed by genetic studies, arrows indicate the direction of the transfer. The geographic incidence of *P. vivax* is indicated in dark grey when high and in light blue when sporadic, based on data from Battle et al. [5] for high-transmission regions and on data from Twohig et al. [71] for low-transmission regions in Africa (*i.e.* reports of all *P. vivax* occurrences in Africa). Map modified from Rougeron et al. [11]

The second point is that *P. vivax* parasites probably colonized the American continent through multiple independent waves of human migrations. A study of the genetic polymorphisms in the 3ʹ end of 18S rRNA gene (expressed during the blood and also the sporozoite stages) and the plastid genome showed genetic differences between Old World and New World *P. vivax* lineages. Moreover, based on genetic polymorphisms, the *P. simium* strain (Fonseca MRA-353) clustered with the Old World *P. vivax* strains. This suggested that *P. simium* and the *P. vivax* populations that currently circulate among humans entered the American continent in at least two separate occasions and very likely from different source populations [21]. In 2003, Carter et al. postulated that *P. vivax* populations arrived in the Americas through two independent human migration waves [22]: the pre-Columbian human migrations from Asia, and then the European colonization and slave trade (Fig. 1). Carter et al. hypothesized that a relapsing *P. vivax* population might have survived the long-range, pre-Columbian oceanic crossing from the Western Pacific to the Americas through a reverse Kon-Tiki route [22]. A study on the genetic variability of 13 microsatellite loci in 108 samples from eight localities in Asia, Africa, South America, and New Guinea showed that Southeast Asian and South American *P. vivax* populations are related to a certain degree [23]. Particularly, *P. vivax* parasites from Venezuela are more closely related to *P. vivax* from Thailand than from Azerbaijan, Ethiopia and Turkey [23]. This suggests that Venezuelan *P. vivax* probably originated from Asia. Another study tried to reconstruct the comprehensive genetic history of *P. vivax* and its association with humans using population genetics and phylogenetic analyses of the mitochondrial genomes of several *P. vivax* populations [24]. Based on these sequencing data, the migration rates among populations, estimated using a maximum likelihood approach, showed that *P. vivax* parasites arrived in the Americas first from Asia and then from Europe [24]. Similarly, a haplotype network analysis suggested that South American *P. vivax* parasites originated from the European/ancient African stock that crossed the Atlantic in the post-Columbus era [18]. Conversely, a haplotype network showed that Melanesia haplotypes form a cluster genetically associated with American *P. vivax*, suggesting an Asian origin of American *P. vivax* [20]. The analysis of complete mitochondrial genomes identified a new and relatively divergent clade of *P. vivax* found mainly in South America, and also other divergent lineages that might have stemmed from independent introductions [25]. Moreover, the analysis of *P. vivax* mitochondrial genomes using a Bayesian phylogeographical method showed genetic similarities between South American and African and also Southeast Asian *P. vivax* populations, supporting a double introduction of *P. vivax* in the Americas [26]. Analysis of four genes suggested multiple origins of *P. vivax*, with two founding populations, one from Eurasia and one from Asia [27]. Similarly, the results obtained in a population genetic study of worldwide *P. vivax* samples (14 microsatellite markers) suggested multiple origins for the American *P. vivax* populations [9]. Indeed, the results demonstrated that South American *P. vivax* populations are more related to Asian populations, whereas Central American *P. vivax* populations (particularly the Mexican population) are more closely related to some African populations (particularly the Mauritanian *P. vivax* population). This suggests again a double origin of *P. vivax* in America one from Africa (or Europe) and the other one from Asia [9] (Fig. 1). Finally, a recent ADMIXTURE clustering analysis based on 1,610,445 single nucleotide polymorphisms (SNP) (i.e. 1 SNP every 13 base pairs of the genome) of previously published *P. vivax* genomes from the American continent (n = 173) provided similar results as those obtained using microsatellite markers (unpublished results). The data allowed the American strains to be grouped into two genetic clusters: (i) one that includes *P. vivax* strains from Peru and Brazil, and (ii) one with *P. vivax* strains from Mexico, El Salvador, Nicaragua, Panama and Colombia. This last result suggests again that *P. vivax* was introduced to the continent through at least two (maybe more) independent waves; however, the exact origins of these successive colonization waves remain unknown. Another point that needs to be investigated is the exact timing of these introductions and the possibility of more ancient, pre-Colombian colonisation of *P. vivax* in the Americas by *P. vivax*. It is possible that *P. vivax* populations were first introduced into the Americas during the pre-Columbian era, and then other introductions occurred from Europe, Asia and Africa. If the original populations were completely replaced or were introgressed with the more recent *P. vivax* strains, then studies based on contemporaneously sampled DNA might be unable to detect these older strains. To evaluate such possibility, it would be important to produce an extensive set of American *P. vivax* genomes to detect tracks of this ancestral population, if the original population has not already been completely replaced. Another option would be to develop more extensive ancient DNA and serological research.

Despite the increasing number of studies investigating the origin(s) of the American *P. vivax* population, the debate is not closed yet, in part because the absence of genetic information on the parasite from the European continent (a possible source of the American *P. vivax*) following its complete eradication in this part of the world. To fill this gap, Gelabert et al. [28] successfully retrieved genetic information of European *P. vivax* isolates extracted from diagnostic slides prepared between 1942 and 1944 [28]. Sequence analysis of these mitochondrial genomes revealed that the European *P. vivax* isolates are genetically related to the most common present-day American haplotypes. This suggests that *P. vivax* entered the American continent during the post-Columbian era, during the colonization by Europeans [28]. Then, Van Dorp et al. [29] managed to extract genomic information from the same microscopic slides prepared in 1944 from an infected patient from Spain, to generate the first genome of a European *P. vivax* strain, named Ebro [29]. Comparison of the Ebro genome with the genomes of *P. vivax* parasites from the rest of the world supports the hypothesis that *P. vivax* might have been introduced during the European colonization of the Americas in the fifteenth century (Fig. 1). Indeed, the Ebro *P. vivax* sample clustered within the American *P. vivax* diversity [29]. However, because *P. vivax* was also probably circulating already in the Americas, such genetic data could be also explained by another scenario in which *P. vivax* was imported into Europe from Americas. Currently, it is not possible to evaluate the direction of this transfer because the number of European *P. vivax* genomes is too small. For this reason, it is important to analyse a larger dataset composed of both European and American *P. vivax* isolates to test different evolutionary scenarios.

All these studies suggest that the Americas were the last land colonized by *P. vivax* parasites. The origin(s) and route(s) of this colonization are still uncertain, although many studies suggest multiple independent introductions in space and time, one from Asia and others from Europe and/or Africa. The discrepancies among studies on *P. vivax* source(s) on the American continent are probably explained by sample availability from South and Central America and also by the potential different sources in Asia, Europe and Africa. Of course, if the introduction of *P. vivax* in the Americas happened through multiple independent waves but some became extinct (living no genetic traces in the current strains), then the only way to reconstruct the real history will be through the analysis of ancient *P. vivax* DNA.

Finally, it is well known that *P. vivax* was endemic throughout much of the United States in the late 19th and early twentieth centuries [30–33]. Since no genetic data is available from these strains, one last aspect that should be investigated is the genetic characterization of these ancient isolates to better understand the origin of *P. vivax* in the Americas.

### A still uncertain timing of the American colonization

Determining the date of *P. vivax* introduction in the New World is not an easy task for several reasons: (i) paleontological and archaeological evidence is rare, (ii) substitution rates are imprecisely known, (iii) there is no general relationship between the TMRCA (Time Most Recent Common Ancestor) and the timing of the introduction of the population under study (due to the possible multiple bottlenecks, founding events, extinctions and/or re-introductions that occurred during the history of the population), (iv) TMRCA estimations could be biased by the heterogeneity of the samples studied (in terms of sampling size and origins), and (iv) *Plasmodium* parasites have complex life cycles that are not adequately reflected in existing coalescent models. For example, the generation time for *P. vivax* is poorly known both because the parasite is difficult to grow in culture and because the parasite is able to cause dormant infections with highly variable times until relapse. This might also vary from one geographic region or population to another.

Despite these difficulties, several studies have tried to estimate the date of *P. vivax* origin in the Americas using genetic methods that exploit genetic variation accumulation in genes and genomes at a rate that is only dependent on the mutation rate (no selection considered) [34]. Using 176 complete mitochondrial genomes, Mu et al. [24] estimated that *P. vivax* populations colonized the Americas between 3800 and 5100 years ago. Jongwutiwes et al. [35], using 106 mitochondrial genomes, obtained estimates between 5200 and 6900 years ago. As these studies used different methods and samples (from different geographic localities), Cornejo et al. [20] combined these two datasets to estimate the TMRCA with two mutation rates. They obtained the same pattern (American *P. vivax* are the youngest parasite populations), and dating estimates from 4500 to 6000 years ago. When they analysed the two datasets separately, they obtained different estimates, probably because of differences in the geographical origin of the parasites included in these datasets [20]. More recently, Rodrigues et al. [26], using Bayesian skyline coalescent models, suggested that *P. vivax* arrived in South America 52,149 years before present (95% highest probability density interval: 29,896 to 60,659 years). Finally, Van Dorp et al. [29], using the complete genome of the Ebro ancient European sample and 15 closely related publicly available genomes sampled over a range of time periods, estimated that the mean age of divergence between Ebro and American *P. vivax* was in the fifteenth century. This is consistent with the introduction of *P. vivax* in the Americas by European colonists.

In conclusion, the timing of *P. vivax* colonization of the Americas remains uncertain. Nevertheless, both archaeological and genetic studies point toward at least two possible independent periods of introduction: one during the pre-Colombian era, probably associated with the introduction of *P. vivax* from Asia, and another during the European colonization, associated with a second wave of *P. vivax* introduction from Europe and/or Africa (Fig. 1). Efforts should now focus on obtaining reliable and converging estimates to understand exactly what happened in the history of modern humans that led to *P. vivax* colonization of the American continent.

### *Plasmodium simium* emergence in the Americas

#### A new species with a neglected geographic distribution

For a long time, *P. simium* was considered as morphologically, genetically and immunologically similar to *P. vivax* [20, 23, 27, 36–38]. Therefore, it was suggested that *P. simium* probably originated from a lateral transfer of *P. vivax* from humans to monkeys. Two studies compared several *P. simium* and human *P. vivax* mitochondrial genomes. The finding that only two SNPs distinguished the two species led some researchers to question the species status of *P. simium* [38, 39]. The recent analysis of the first *P. simium* complete genomes from humans and from monkeys showed a clear genetic separation without genetic exchange (absence of genetic hybrids) between *P. vivax* and *P. simium*, a result in agreement with the existence of two distinct *Plasmodium* species [40].

*Plasmodium simium* is a parasite of various species of Platyrrhini monkeys (i.e. New World monkeys) and it was thought that it naturally infected only hosts of the Atlantic forest from Southeast and South Brazil. Indeed, *P. simium* was discovered by da Fonseca in 1951, by microscopy observation of blood smear slides of *Alouatta clamitans* collected in the Atlantic Forest close to Sao Paulo, Brazil [41]. This was the first morphological description of *P. simium* erythrocytic phase. Then, in the 1960s, Deane et al. [37] described the presence of *P. simium* in another monkey species, *Alouatta fusca* (howler monkeys), that lives in the forest reserve of Horto Florestal da Cantareira. They suggested that *Anopheles* (*Kerteszia*) *cruzii* was the vector responsible of *P. simium* transmission [37]. In the following years, several studies reported natural infections by *P. simium* in a variety of monkeys, including *Alouatta caraya*, *Brachyteles arachnoides, Cebus appela*, *Aotus trivirgatus*, *Aotus nancymai*, *Callicebus nigrifrons* [42–52]. *Plasmodium simium* was thus considered to be geographically restricted to the Brazilian Atlantic forest until 2019, when Rondon et al. [53] studied the prevalence of *Plasmodium* parasites in non-human primates (NHPs) of five forest patches in Colombia with the aim of inferring the risk of zoonotic malaria transmission from NHPs to humans. Their discovery of *P. simium* parasites in three NHPs species in Colombia (*Ateles hybridus*, *Cebus versicolor,* and *Alouatta seniculus*) [53] challenged the idea that *P. simium* was only restricted to Brazil, and suggested that its geographic distribution had been largely underestimated. A question then arises: are these two *P. simium* populations corresponding to two distinct species of *Plasmodium* infecting New World monkeys? Currently only Brazilian *P. simium* strains have been genetically characterized. Studies should thus in the future evaluate the natural host range diversity and geographic distribution of *P. simium* in the Americas, as well as the genetic structure of these new distinct *P. simium* populations, in order to unravel the evolutionary history of this parasite in the American continent.

#### Origin and direction of the transfer

**Fig. 2:**
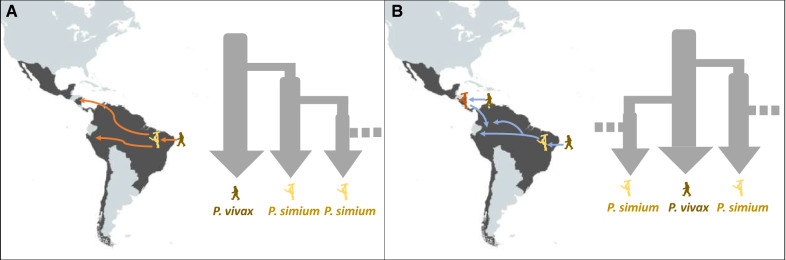
Alternative scenarios for the origin and routes of invasion of America by *P. simium*. **A** The monkey *P. simium* clade derived from the human American *P. vivax* clade. Once local American monkeys started to be infected, *P. simium* expanded and spread through local monkey populations. **B** The monkey *P. simium* clade derived from two independent human American *P. vivax* populations that arrived in America

The close genetic similarity between human *P. vivax* and primate *P. simium* suggests the recent occurrence of host transfers; however, for long time it was not known whether the transfers were from humans to New World monkeys or vice versa, and when they occurred. A phylogenetic study based on the circumsporozoite protein (CSP) of *P. vivax* and *P. simium* proposed that the switch occurred from a Catarrhini primate to a Platyrrhini primate, thus suggesting that *P. simium* originated from *P. vivax* [54]. Another study based on four nuclear genes investigated the phylogenies, haplotype networks, positive selection and genetic diversity of *P. vivax* and *P. simium* strains [27]. This analysis demonstrated that human *P. vivax* and primate *P. simium* recently derived from one or the other. Unfortunately, the direction of the host transfer was not inferred due to the scarcity of available strains from the primate parasites. By comparing the genetic diversity in *P. vivax* and *P. simium* populations, one could test explicitly the most likely evolutionary scenario from which these two species evolved. Following this hypothesis, several works showed that human *P. vivax* strains display higher genetic diversities than *P. simium* strains. Specifically, analyses of the Duffy binding protein (DBP) genes showed that *dbp* gene variability is lower in *P. simium* than *P. vivax* [51]. The analysis of mitochondrial genomes also found lower genetic diversity in *P. simium* than *P. vivax.* Therefore, the authors suggested a recent human-to-monkey transfer that occurred in Brazil [26] (Fig. 2A). Moreover, based on the small subunit (SSU) rRNA gene analysis, the authors observed that a *P. simium* strain (U69605) clustered with *P. cynomolgi,* whereas the other *P. simium* strain (AY579415) clustered with the *P. vivax* isolates. This led to the hypothesis that the host transfer between humans and New World monkeys in Brazil occurred not once, but twice during the history of this pathogen. A plausible scenario is that *P. vivax* was introduced first in the New World monkeys following the Europeans’ arrival in the American continent, and then from another source, as suggested also by a previous study [55] (Fig. 2B). Indeed, this earlier work on *csp* gene polymorphisms suggests that at least two host transfers occurred between humans and New World monkeys. Similarly, in 2001 Li et al. [21] suggested that *P. vivax* arrived in the Americas through two distinct (European and/or African) waves during the colonization period that led to the current *P. vivax* populations of the Americas, and a Southeast Asian wave that also founded the current *P. simium* populations. Similarly, Carter et al. [22] speculated that an ancient Asian stock of *P. vivax* arrived in the Americas to give rise to *P. simium*. In 2010, Mourier and colleagues [40] sequenced the first nuclear genomes of *P. simium* isolates that infect monkeys and also humans in Brazil. They found that *P. vivax* strains are characterized by significantly higher genetic diversity compared with *P. simium* strains. This suggested that *P. simium* probably evolved from a *P. vivax* transfer from humans to American monkeys [40]. More recently, de Oliveira et al. [56] used a genomic approach to show that the mean genetic diversity of *P. simium* strains in Brazil is approximately three times lower than that of American *P. vivax* populations. They suggested that *P. vivax* isolates arrived in Brazil from southern Europe and Africa, became adapted to local vector species (*Kerteszia* mosquitoes), and jumped from humans to sylvatic monkeys [56].

The transfer direction from human to monkey seems to be widely accepted in the community; however, the possibility of several transfers from different origins is still debated, partly because most of the published studies only analysed *P. simium* strains collected in Brazil. The detection of *P. simium* circulating in various monkey species in Colombia reopens the question of *P. simium* origin: (i) a single transfer of *P. vivax* on the American continent (i.e. Brazil) from humans to monkeys to give rise to *P. simium* that then diffused among the monkey populations of different American countries (Fig. 2A); or (ii) several *P. vivax* independent transfers of *P. vivax* from humans to monkeys in different places in the Americas, giving rise to several genetically differentiated *P. simium* strains (i.e. in Brazil, in Colombia or Central America). All these questions need to be addressed by generating new genomic data using *P. simium* isolates from other American countries and form different monkey species.

#### Evolutionary adaptation to local American environmental conditions

When *P. vivax* arrived in the Americas (from Europe, Africa and/or Asia), it found new environmental conditions: new vector species, new human populations, and also new host species following its transfer to New World monkeys. How did it adapt to these new environmental conditions? Did these adaptations to new environments involve molecular adaptations?

#### *Plasmodium vivax* adaptation to new vectors

In the different regions of the New World, distinct anopheles species transmit *Plasmodium* parasites [57, 58]. In North America (USA, Canada and north of Mexico), the dominant vectors are *Anopheles freeborni* in the west, and *Anopheles quadrimaculatus* in the East. In Central America, the main vectors are *Anopheles albimanus* and *Anopheles pseudopunctipennis*. *Anopheles (Nyssorhinchus*) *darlingi* is the main vector in South America, but with a more localized and patchy distribution. Other species also are involved in the parasite transmission, such as species of the complex *Anopheles albitarsis *sensu lato (*s.l.*), and *Anopheles aquasalis.* The latter is not a very good vector, but it is the ‘dominant’ species in coastal areas of Central and South America because of its ability to use saline habitats for larval development [57, 58].

The New World *Anopheles* vectors are genetically very divergent from the Old World vectors. Indeed, it is thought that they diverged 95 million years ago, following the separation of the American continent from what is now Africa [59]. Therefore, it is not surprising that the New World and the Old World vectors present many genetic, ecological and behavioural differences. These differences might have exerted strong selective pressures on *P. vivax* parasites (as they did on *P. falciparum—*see [1] for a review) that may have influenced their local evolution [60, 61].

Although the number of studies is limited, the available evidence suggests that *P. vivax* adapted to the local vector species to ensure its transmission [21]. Comparison of the infectivity of several New World and Old World *P. vivax* strains using the New World *An. albimanus* vector showed much higher infection rates by American than Old World *P. vivax* strains. Conversely, infectivity to *An. freeborni*, an American mosquito species that lives outside the range of malaria transmission on the continent, was comparable between New World and Old World *P. vivax* strains. These results indicate that New World *P. vivax* strains adapted to the local mosquitoes (particularly *An. albimanus*) to ensure their transmission.

Most studies on *P. vivax* adaptation to the mosquito vectors in South America were performed in the region of Chiapas in southern of Mexico. In this region, transmission at low altitude (< 100 m) is carried out by *An. albimanus*, and at higher altitude by *An. pseudopunctipennis*. The first studies performed in this region found that the parasite populations living at low altitude and in the foothills are genetically different [62]. Specifically, in the coastal plains, all *P. vivax* human infections were caused by *P. vivax* harbouring the CSP variant VK210 (a variant defined by the nature and number of repeat units in the *csp* gene). Conversely, in the foothills, 50% of the infections were caused by *P. vivax* harbouring the CSP variant VK247. This led to the hypothesis that *P. vivax* harbouring the variant VK247 was transmitted preferentially by *An. pseudopunctipennis*. This hypothesis was confirmed a few years later by comparing the infectivity of the *P. vivax* strains harbouring the V247 and V210 variants to the two local vector species [63]. Indeed, *An. albimanus* was more susceptible to *P. vivax* harbouring the VK210 variant, and *An. pseudopunctipennis* to *P. vivax* harbouring the VK247 variant. Nevertheless, the authors noted that their results could be biased because all *P. vivax* strains carrying the VK210 variant were from the coastal plains, whereas those harbouring the VK217 variant were all from the foothills. Therefore, the *csp* gene variation might not be implicated in the adaptation to the different mosquito species, but might be correlated to another polymorphism if populations from below and above 100 m were genetically different. Subsequent studies questioned the role of CSP in the adaptation to the different mosquito species. It was shown that *P. vivax* destruction in the unsusceptible mosquito species (i.e. *An. pseudopunctipennis* for *P. vivax* harbouring the V210 variant and *An. albimanus* for *P. vivax* harbouring the V247 variant) occurred before CSP expression [64, 65]. Furthermore, in Colombia, *P. vivax* harbouring VK247 variant was more frequently found in *An. pseudopunctipennis* than in *An. albimanus* (and the opposite for *P. vivax* harbouring the VK210 variant)*.* In 2008, Joy et al. [66] analysed the population genetic structure of *P. vivax* populations collected in Mexico using a set of microsatellite markers. They demonstrated that in this region, *P. vivax* is genetically subdivided into three distinct populations and that this subdivision largely mirrors the distribution of the two mosquito species (and hence the different CSP variants). They also showed that each population was locally adapted to their respective mosquito vector because each population optimally infected its sympatric mosquito species. Their results suggested that the relationship between CSP polymorphisms and vector compatibility could be spurious, and simply due to the genetic subdivision between *P. vivax* populations that concerned all loci in the genomes. Subsequent studies analysed the polymorphisms of other proteins, especially ookinete-specific proteins (e.g. Pf25/pf27 [67]) and genes responsible for the parasite penetration in the mosquito midgut, as determinants of the differences in parasite infectivity between mosquito species. These studies were performed using only local mosquito populations [67] or populations from the entire Mexico [68], and they detected an association between the parasite genotype and its compatibility with a mosquito species. Like for CSP, these studies might have highlighted a spurious association between candidate loci and phenotype due to the parasite population genetic structure. Indeed, the parasite strains used in these studies were always from the Chiapas region around Tapachula, in Mexico.

Additional indirect evidence of selection by the mosquito vectors involved in *P. vivax* transmission in America was obtained from genetic/genomic studies. In [19], the genomes of several hundred *P. vivax* strains, mainly from Asia and South America, were sequenced. The analysis of genetic divergence in the different populations highlighted the presence of higher divergence on chromosome 12 between New World and Old World populations compared with the divergence between Thailand and Papua New Guinea populations. The locally elevated differentiation along the genome (which is a signal of local adaptation) was centred on a region including the *Pvs47* gene involved in the parasite-vector interaction. Moreover, this region had lower haplotype diversity (compared with the rest of the genome) but no higher local linkage disequilibrium (LD). This suggests that this locally-selected genomic region had been subject to positive selection (e.g., selective sweep). However, because the region is not characterized by increased LD, this means that selection was either not very recent (recombination had time to reduce LD locally) or occurred on a polymorphism that was already frequent in the population (soft sweep). Interestingly, similar patterns of selection were observed in the orthologous gene in *P. falciparum* (*Pfs47*) that plays a role in the parasite evasion from the mosquito immune response. This suggests that, like for *P. falciparum*, *Pvs47* haplotypes/polymorphisms could be differentially selected by different vector species. It also indicates that the interaction of *Pv47* haplotypes with specific haplotypes of its target in the mosquito might have been critical for the parasite’s adaptation to new vectors. The same result has been obtained in the study of Benavente et al. [69], that showed mutations in genes (pvs47, pvs48/45) associated with mosquito stages in South American *P. vivax* populations. Another gene, merozoite apical erythrocyte binding ligand (*MAEBL*) that binds to red blood cells, also plays a role in the infection of the mosquito salivary glands by sporozoites. This suggests that mosquito species may have shaped the evolution of this protein in the Americas.

It seems now quite certain that *P. vivax* adapted to the specific vectors found in South America and their specificity in south America. Genetic and genomic studies have allowed us to identify certain genes that might have played a role in this adaptation but there is still a lot to learn regarding the genetic determinants of this adaptation.

#### Adaptation of *Plasmodium vivax* to new human populations

The current human populations in the American continent are diverse. They are characterized by a recent history of admixture among populations from different regions of the world (native Americans, Africans, Europeans, and Asians).

Very few studies have explored *P. vivax* adaptation to the human populations in South America. The only evidence that the local human populations and their specific features may have influenced *P. vivax* evolution in South America came from studies that used population genetic statistical methods to detect signals of selection in the available genome sequences. These studies produced a list of genes, among which some are known to be expressed in human stages and could therefore be good candidates in the adaptation to humans [19, 69, 70]. This is for instance the case for *maebl* gene, *msp3*, *msp4*, *msp5*, *msp10* and reticulocyte binding protein 3 (*rpb3*). Some of these proteins (i.e. the MAEBL protein) are known to be involved in the invasion of the host red blood cells. These proteins can be under two distinct types of selective pressures exerted by the human environment (well characterized in *P. falciparum*): (1) the immune system because most of these proteins are surface proteins recognized by the host immune system and tend to be under balancing selection; and (2) the host receptor (if the parasite protein is a ligand) in which sequence variations can change the specificity for different ligand variants in the parasite.

Evidence of adaptation of the parasite to the American human populations is still very scarce. More detailed studies of the parasite population genomic characteristics in America in comparison to population from the source populations (in Asia, Europe and Africa) could provide a first set of candidate genes that responded to the specific selective pressures imposed by the American human populations to the parasite.

#### Adaptation of *Plasmodium simium*

As observed for *P. vivax* adaptation to new Anopheles species, the *Pvs47* gene was identified as involved also in *P. simium* adaptation to local vectors. Indeed, de Oliveira et al. [56] investigated the infectivity of *P. simium* from Brazil to Anopheles *Nyssorhinchus* (the dominant vector species in the interior of the continent) and *Anopheles Kerteszia* (a more coastal species) and analysed the adaptive genetic changes. According to their hypothesis, *P. vivax* arrived in the American continent from a European/African stock that evolved locally to infect anopheles *Kerteszia* species and then local monkey species. They found that *P. simium Pvs47* sequences clustered with those of American *P. vivax*, and suggested that this was a consequence of the selection imposed by adaptation to local *Kerteszia* vectors along the Atlantic coasts in South America.

Regarding *P. simium* adaptation to different American primate species and human populations, some very recent studies identified the first molecular signatures of local adaptation. Mourier et al. [40] published the first *P. simium* genomes, and showed some structural variation in the Duffy-binding protein 1 (*DBP1*) and *RBP2a* genes. They found that the invasion gene *DBP1* of *P. simium* contains a 115 base-pairs (bp) deletion in its coding region. They also detected another 1 kb deletion in the coding region of the *RPB2a* gene [40]. Similarly, de Oliveira et al. [56] reported that *P. simium* PvRBP2a sequence is characterized by a 1004 amino acid deletion in exon 2 that preserves the reading frame, but removes 44% of the coding sequence [56]. The authors suggested that such large modifications probably affect the red blood cell binding affinity, thus favouring other still unknown ligands in the adaptation to infect red blood cells of American monkeys and humans.

Signatures of *P. vivax* local adaptation have been identified in the *PvRBP2a* and *DBP1* genes; however, the infection efficiency in monkeys and humans is unknown. Functional assays should be developed to evaluate the interaction of such ligands with red blood cells. Moreover, the genomes of *P. simium* strains collected in other geographic localities, such as Colombia, should also be investigated.

## Conclusion

The increasing amount of genetic and genomic information on *P. vivax* parasites has led to major advances in understanding their origin and dispersal through the world. Currently, it is accepted that the American continent was probably the last piece of land colonized by *P. vivax* parasites. However, the origins and route of dispersal are still puzzling, and multiple origins from Asia and/or Europe and/or Africa have been suggested. Only genome sequences from more American and historical ancient European *P. vivax* parasites will help to reconstruct the evolutionary origin and spread of this malaria parasite.

Concerning the origins of *P. simium*, the research community acknowledges that it occurred through a *P. vivax* transfer from humans to local monkey species on the American continent. Nevertheless, the possibility of several transfers from different origins has not been confirmed yet, partly because most of the published studies only analysed *P. simium* strains collected from Brazil.

Finally, very little is known about the mechanisms that allowed *P. vivax* adaptation to humans and mosquitoes and to the different environments in the New World. For *P. simium*, the very limited genomic data and only from Brazil undeniably limit our understanding of its genetic adaptations in this new environment. Future studies should focus on the generation of new genomic data from different American countries (Central and South America) and on the development of functional approaches to validate the genetic features that might influence the ecological success of infection and thus the fitness of these *Plasmodium* species in different environments.

## Data Availability

Not applicable.

## References

[1] WHO. World malaria report 2020. Geneva, World Health Organization, 2020. https://www.who.int/publications-detail-redirect/9789240015791. Accessed 26 Oct 2020.

[2] Howes RE, Battle KE, Mendis KN, Smith DL, Cibulskis RE, Baird JK (2016). Global epidemiology of *Plasmodium vivax*. Am J Trop Med Hyg.

[3] Carlton JM, Das A, Escalante AA (2013). Genomics, population genetics and evolutionary history of *Plasmodium vivax*. Adv Parasitol.

[4] Carlton JM, Adams JH, Silva JC, Bidwell SL, Lorenzi H, Caler E (2008). Comparative genomics of the neglected human malaria parasite *Plasmodium vivax*. Nature.

[5] Battle KE, Lucas TCD, Nguyen M, Howes RE, Nandi AK, Twohig KA (2019). Mapping the global endemicity and clinical burden of *Plasmodium vivax*, 2000–17: a spatial and temporal modelling study. Lancet.

[6] Flegontov P, Altınışık NE, Changmai P, Rohland N, Mallick S, Adamski N (2019). Palaeo-Eskimo genetic ancestry and the peopling of Chukotka and North America. Nature.

[7] Yalcindag E, Elguero E, Arnathau C, Durand P, Akiana J, Anderson TJ (2012). Multiple independent introductions of *Plasmodium falciparum* in South America. Proc Natl Acad Sci USA.

[8] Pan American Health Organization (PAHO) ILAE IBE//International League Against Epilepsy. https://www.ilae.org/about-ilae/public-policy-and-advocacy/pan-american-health-organization-paho. Accessed 26 Oct 2021.

[9] Rougeron V, Elguero E, Arnathau C, Acuña Hidalgo B, Durand P, Houze S (2020). Human *Plasmodium vivax* diversity, population structure and evolutionary origin. PLoS Negl Trop Dis.

[10] Daron J, Boissière A, Boundenga L, Ngoubangoye B, Houze S, Arnathau C (2021). Population genomic evidence of *Plasmodium vivax* Southeast Asian origin. Sci Adv.

[11] Rougeron V, Boundenga L, Arnathau C, Durand P, Renaud F, Prugnolle F (2022). A population genetic perspective on the origin, spread and adaptation of the human malaria agents *Plasmodium falciparum* and *Plasmodium vivax*. FEMS Microbiology Rev.

[12] Tito RY, Macmil S, Wiley G, Najar F, Cleeland L, Qu C (2008). Phylotyping and functional analysis of two ancient human microbiomes. PLoS ONE.

[13] Wiscovitch-Russo R, Narganes-Stordes Y, Cano RJ, Toranzos GA (2019). Origin of the new world *Plasmodium vivax*: facts and new approaches. Int Microbiol.

[14] Gerszten E, Allison MJ, Maguire B (2012). Paleopathology in South American mummies: a review and new findings. Pathobiology.

[15] DeGiorgio M, Jakobsson M, Rosenberg NA (2009). Explaining worldwide patterns of human genetic variation using a coalescent-based serial founder model of migration outward from Africa. Proc Natl Acad Sci USA.

[16] Tanabe K, Mita T, Jombart T, Eriksson A, Horibe S, Palacpac N (2010). *Plasmodium falciparum* accompanied the human expansion out of Africa. Curr Biol.

[17] Imwong M, Nair S, Pukrittayakamee S, Sudimack D, Williams JT, Mayxay M (2007). Contrasting genetic structure in *Plasmodium vivax* populations from Asia and South America. Int J Parasitol.

[18] Culleton R, Coban C, Zeyrek FY, Cravo P, Kaneko A, Randrianarivelojosia M (2011). The origins of African *Plasmodium vivax*; insights from mitochondrial genome sequencing. PLoS ONE.

[19] Hupalo DN, Luo Z, Melnikov A, Sutton PL, Rogov P, Escalante A (2016). Population genomics studies identify signatures of global dispersal and drug resistance in *Plasmodium vivax*. Nat Genet.

[20] Cornejo OE, Escalante AA (2006). The origin and age of *Plasmodium vivax*. Trends iParasitol.

[21] Li J, Collins WE, Wirtz RA, Rathore D, Lal A, McCutchan TF (2001). Geographic subdivision of the range of the malaria parasite *Plasmodium vivax*. Emerg Infect Dis.

[22] Carter R (2003). Speculations on the origins of *Plasmodium vivax* malaria. Trends Parasitol.

[23] Leclerc MC, Durand P, Gauthier C, Patot S, Billotte N, Menegon M (2004). Meager genetic variability of the human malaria agent *Plasmodium vivax*. Proc Natl Acad Sci USA.

[24] Mu J, Joy DA, Duan J, Huang Y, Carlton J, Walker J (2005). Host switch leads to emergence of *Plasmodium vivax* malaria in humans. Mol Biol Evol.

[25] Taylor JE, Pacheco MA, Bacon DJ, Beg MA, Machado RL, Fairhurst RM (2013). The evolutionary history of *Plasmodium vivax* as inferred from mitochondrial genomes: parasite genetic diversity in the Americas. Mol Biol Evol.

[26] Rodrigues PT, Valdivia HO, de Oliveira TC, Alves JMP, Duarte AMRC, Cerutti-Junior C (2018). Human migration and the spread of malaria parasites to the New World. Sci Rep.

[27] Tazi L, Ayala FJ (2011). Unresolved direction of host transfer of *Plasmodium vivax* v. *P*. *simium* and *P*. *malariae* v. *P*. *brasilianum*. Infect Genet Evol.

[28] Gelabert P, Sandoval-Velasco M, Olalde I, Fregel R, Rieux A, Escosa R (2016). Mitochondrial DNA from the eradicated European *Plasmodium**vivax* and *P*. *falciparum* from 70-year-old slides from the Ebro Delta in Spain. Proc Natl Acad Sci USA.

[29] van Dorp L, Gelabert P, Rieux A, de Manuel M, de Dios T, Gopalakrishnan S (2020). *Plasmodium vivax* malaria viewed through the lens of an eradicated European strain. Mol Biol Evol.

[30] Maldonado YA, Nahlen BL, Roberto RR, Ginsberg M, Orellana E, Mizrahi M (1990). Transmission of *Plasmodium vivax* malaria in San Diego County, California, 1986. Am J Trop Med Hyg.

[31] Zucker JR (1996). Changing patterns of autochthonous malaria transmission in the United States: a review of recent outbreaks. Emerg Infect Dis.

[32] Sunstrum J, Elliott LJ, Barat LM, Walker ED, Zucker JR (2001). Probable autochthonous *Plasmodium vivax* malaria transmission in Michigan: case report and epidemiological investigation. Am J Trop Med Hyg.

[33] Dye-Braumuller KC, Kanyangarara M (2021). Malaria in the USA: how vulnerable are we to future outbreaks?. Curr Trop Med Rep.

[34] Bensch S, Hellgren O, Križanauskiene A, Palinauskas V, Valkiunas G, Outlaw D (2013). How can we determine the molecular clock of malaria parasites?. Trends Parasitol.

[35] Jongwutiwes S, Putaporntip C, Iwasaki T, Ferreira MU, Kanbara H, Hughes AL (2005). Mitochondrial genome sequences support ancient population expansion in *Plasmodium vivax*. Mol Biol Evol.

[36] Ayala FJ, Escalante AA, Rich SM (1999). Evolution of *Plasmodium* and the recent origin of the world populations of *Plasmodium falciparum*. Parasitologia.

[37] Deane LM, Deane MP, Neto JF, Organization WH (1966). Studies on transmission of simian malaria and on a natural infection of man with *Plasmodium simium*, in Brazil. Bull World Health Organ.

[38] Brasil P, Zalis MG, de Pina-Costa A, Siqueira AM, Júnior CB, Silva S (2017). Outbreak of human malaria caused by *Plasmodium simium* in the Atlantic Forest in Rio de Janeiro: a molecular epidemiological investigation. Lancet Glob Health.

[39] Alvarenga DAM, Pina-Costa A, Bianco C, Moreira SB, Brasil P, Pissinatti A (2017). New potential *Plasmodium brasilianum* hosts: tamarin and marmoset monkeys (family Callitrichidae). Malar J.

[40] Mourier T, de Alvarenga DAM, Kaushik A, de Pina-Costa A, Douvropoulou O, Guan Q (2021). The genome of the zoonotic malaria parasite *Plasmodium simium* reveals adaptations to host switching. BMC Biol.

[41] Da Fonseca F (1951). [Plasmodium of a primate of Brazil] (in portugese). Mem Inst Oswaldo Cruz.

[42] Deane LM, Ferreira Neto JA (1969). [Presence of *Plasmodium brasilianum* in monkeys from the Federal Territory of Amapa, Brazil](in Portuguese). Rev Inst Med Trop Sao Paulo.

[43] Collins WE, Contacos PG, Guinn EG, Skinner JC (1973). *Plasmodium simium* in the *Aotus trivirgatus* monkey. J Parasitol.

[44] Collins WE, Warren M, Contacos PG, Skinner JC, Richardson BB (1979). Infectivity of *Plasmodium simium* to *Aotus trivirgatus* monkeys and different anophelines. J Parasitol.

[45] Collins WE, Skinner JC, Pappaioanou M, Broderson JR, Ma NSF, Stanfill PS (1987). Transmission of *Plasmodium**simium* to *Aotus**nancymai*, *A*. *vociferans*, *A*. *azarae**boliviensis*, and *Saimiri**sciureus**boliviensis* Monkeys. J Parasitol.

[46] Deane LM (1992). Simian malaria in Brazil. Mem Inst Oswaldo Cruz.

[47] Bueno MG, Rohe F, Kirchgatter K, Di Santi SMF, Guimarães LO, Witte CL (2013). Survey of *Plasmodium* spp. in free-ranging neotropical primates from the Brazilian Amazon region impacted by anthropogenic actions. EcoHealth.

[48] Araújo MS, Messias MR, Figueiró MR, Gil LHS, Probst CM, Vidal NM (2013). Natural *Plasmodium* infection in monkeys in the state of Rondônia (Brazilian Western Amazon). Malar J.

[49] Costa DC, da Cunha VP, de Assis GMP, de Souza Junior JC, Hirano ZMB, de Arruda ME (2014). *Plasmodium simium/Plasmodium vivax* infections in southern brown howler monkeys from the Atlantic Forest. Mem Inst Oswaldo Cruz.

[50] de Alvarenga DAM, de Pina-Costa A, de Sousa TN, Pissinatti A, Zalis MG, Suaréz-Mutis MC (2015). Simian malaria in the Brazilian Atlantic forest: first description of natural infection of capuchin monkeys (*Cebinae* subfamily) by *Plasmodium simium*. Malar J.

[51] Camargos Costa D, de Pereira Assis GM, de Souza Silva FA, Araújo FC, de Souza Junior JC, Braga Hirano ZM (2015). *Plasmodium simium*, a *Plasmodium vivax*-related malaria parasite: genetic variability of Duffy binding protein II and the Duffy antigen/receptor for chemokines. PLoS ONE.

[52] de Assis GMP, de Alvarenga DAM, Costa DC, de Souza JC, Hirano ZMB, Kano FS (2016). Detection of *Plasmodium* in faeces of the New World primate *Alouatta clamitans*. Mem Inst Oswaldo Cruz.

[53] Rondón S, León C, Link A, González C (2019). Prevalence of *Plasmodium* parasites in non-human primates and mosquitoes in areas with different degrees of fragmentation in Colombia. Malar J.

[54] Escalante AA, Freeland DE, Collins WE, Lal AA (1998). The evolution of primate malaria parasites based on the gene encoding cytochrome b from the linear mitochondrial genome. Proc Natl Acad Sci USA.

[55] Lim CS, Tazi L, Ayala FJ (2005). *Plasmodium vivax*: recent world expansion and genetic identity to *Plasmodium**simium*. Proc Natl Acad Sci USA.

[56] de Oliveira TC, Rodrigues PT, Duarte AMRC, Rona LDP, Ferreira MU (2021). Ongoing host-shift speciation in *Plasmodium simium*. Trends Parasitol.

[57] Sinka ME, Bangs MJ, Manguin S, Rubio-Palis Y, Chareonviriyaphap T, Coetzee M (2012). A global map of dominant malaria vectors. Parasit Vectors.

[58] Sinka ME, Rubio-Palis Y, Manguin S, Patil AP, Temperley WH, Gething PW (2011). Erratum: the dominant *Anopheles* vectors of human malaria in the Americas: occurrence data, distribution maps and bionomic précis (Parasit Vectors 2010;3:72). Parasit Vectors.

[59] Neafsey DE, Waterhouse RM, Abai MR, Aganezov SS, Alekseyev MA, Allen JE (2015). Mosquito genomics. Highly evolvable malaria vectors: the genomes of 16 *Anopheles* mosquitoes. Science.

[60] Molina-Cruz A, Zilversmit MM, Neafsey DE, Hartl DL, Barillas-Mury C (2016). Mosquito vectors and the globalization of *Plasmodium falciparum* malaria. Annu Rev Genet.

[61] Molina-Cruz A, Barillas-Mury C (2014). The remarkable journey of adaptation of the *Plasmodium falciparum* malaria parasite to New World anopheline mosquitoes. Mem Inst Oswaldo Cruz.

[62] Kain KC, Wirtz RA, Fernandez I, Franke ED, Rodriguez MH, Lanar DE (1992). Serologic and genetic characterization of *Plasmodium vivax* from whole blood-impregnated filter paper discs. Am J Trop Med Hyg.

[63] Gonzalez-Ceron L, Rodriguez MH, Nettel JC, Villarreal C, Kain KC, Hernandez JE (1999). Differential susceptibilities of *Anopheles albimanus* and *Anopheles pseudopunctipennis* to infections with coindigenous *Plasmodium vivax* variants VK210 and VK247 in Southern Mexico. Infect Immun.

[64] Gonzalez-Ceron L, Rodriguez MH, Santillan F, Chavez B, Nettel JA, Hernandez-Avila JE (2001). *Plasmodium vivax*: ookinete destruction and oocyst development arrest are responsible for *Anopheles albimanus* resistance to circumsporozoite phenotype VK247 parasites. Exp Parasitol.

[65] Gonzalez-Ceron L, Rodriguez MH, Chavez-Munguia B, Santillan F, Nettel JA, Hernandez-Avila JE (2007). *Plasmodium vivax*: impaired escape of Vk210 phenotype ookinetes from the midgut blood bolus of *Anopheles pseudopunctipennis*. Exp Parasitol.

[66] Joy DA, Gonzalez-Ceron L, Carlton JM, Gueye A, Fay M, McCutchan TF (2008). Local adaptation and vector-mediated population structure in *Plasmodium**vivax* malaria. Mol Biol Evol.

[67] González-Cerón L, Alvarado-Delgado A, Martínez-Barnetche J, Rodríguez MH, Ovilla-Muñoz M, Pérez F (2010). Sequence variation of ookinete surface proteins Pvs25 and Pvs28 of *Plasmodium vivax* isolates from Southern Mexico and their association to local anophelines infectivity. Infect Genet Evol.

[68] González Cerón L, Rodríguez MH, Nettel Cruz JA, Hernández Ávila JE, Malo García IR, Santillán Valenzuela F (2019). *Plasmodium**vivax* CSP-Pvs25 variants from southern Mexico produce distinct patterns of infectivity for *Anopheles**albimanus* versus *An*. *pseudopunctipennis*, in each case independent of geographical origin. Parasit Vectors.

[69] Benavente ED, Manko E, Phelan J, Campos M, Nolder D, Fernandez D (2021). Distinctive genetic structure and selection patterns in *Plasmodium vivax* from South Asia and East Africa. Nat Commun.

[70] Diez Benavente E, Ward Z, Chan W, Mohareb FR, Sutherland CJ, Roper C (2017). Genomic variation in *Plasmodium vivax* malaria reveals regions under selective pressure. PLoS ONE.

[71] Twohig KA, Pfeffer DA, Baird JK, Price RN, Zimmerman PA, Hay SI (2019). Growing evidence of *Plasmodium vivax* across malaria-endemic Africa. PLoS Negl Trop Dis.

